# Laughter

**Published:** 1910-01

**Authors:** 


### Laughter.

Laughter is the adipose tissue which softens man's points of contact with a hard world. It is his spontaneous concurrence with God's original verdict concerning the goodness of the earth, and is his insouciant challenge to fate. It does not deny the fact of the inherent pathos of life, nor does it close its eyes to the presence of the wrong. It is the soul of optimism, believing that evil is ultimately impotent. It holds that sham can lift no lance it can not shiver, that hypocrisy can bear no shield it can not cleave, nor bigotry find refuge behind rampart it can not scale. It is not unmindful that the march of the race has been along a thoroughfare paved with the bones of mothers' sons, sprinkled with women's tears, and having as its mile stones the crosses of the saviors of men. It believes that these things are but incidents of progress, the necessary growing pains of a youthful world. It thinks in terms of eons, and is the master of perspective. It gives to man that point of vantage whence, though his way leads through the thicket growth, he is yet enabled to perceive the pleasing symmetry of the forest. With its squint-eyed vision it sees life more clearly because it sees its awry.

Laughter is dynamic common sense. It is kinetic faith, hope and charity. In it, as in the poet's flower from the crannied wall, lies implicit all the wisdom of the sages and the seers.—*John E. Rosser, Austin, Texas, in the Cosmopolitan, February,* 1910.

---

God and Bwana Tumbo (Roosevelt) are at variance about big families. We are told that Jesus was God's only begotten son. God believes in quality; Bwana wants numbers.—*The Philistine.*

